# Antiproliferative Rapeseed Defatted Meal Protein and Their Hydrolysates on MCF-7 Breast Cancer Cells and Human Fibroblasts

**DOI:** 10.3390/foods10020309

**Published:** 2021-02-03

**Authors:** Romina L. Ferrero, Carmen Soto-Maldonado, Caroline Weinstein-Oppenheimer, Zaida Cabrera-Muñoz, María Elvira Zúñiga-Hansen

**Affiliations:** 1 Escuela de Ingeniería Bioquímica, Pontificia Universidad Católica de Valparaíso, Av. Brasil 2085, Valparaíso 2362803, Chile; romi.ferrero@hotmail.com (R.L.F.); carmensoto@creas.cl (C.S.-M.); zaida.cabrera@pucv.cl (Z.C.-M.); 2 Centro Regional de Estudio en Alimentos Saludables, R17A10001, Av. Universidad 330, Curauma, Valparaíso 2373223, Chile; 3 Escuela de Química y Farmacia, Facultad de Farmacia, Universidad de Valparaíso, Gran Bretaña 1093, Playa Ancha, Valparaíso 2360102, Chile; caroline.weinstein@uv.cl; 4 Centro de Investigación Farmacopea Chilena, Santa Marta 183, Playa Ancha, Valparaíso 2360134, Chile

**Keywords:** bioactive peptides, antiproliferative activity, breast cancer, anticancer, meal rapeseed

## Abstract

Defatted rapeseed meal (DRM) is a sub-valorized agro-industrial by-product, with a high protein content whose peptides could have potential anticancer activity against cancer cell lines. The objective of the present study is to obtain an enzymatic hydrolysate of rapeseed protein that inhibits proliferation on a breast cancer cell line (MCF-7), but not healthy human fibroblast cells. The DRM was solubilized in an alkaline medium to obtain an alkaline rapeseed extract (RAE). Acid precipitation of the proteins contained in RAE recovered a rapeseed protein isolate (RPI). To produce protein hydrolysates, two alkaline protease and different enzyme/substrate ratios were used. All the protein hydrolysates showed antiproliferative activity on MCF-7 cells. However, only the hydrolysate recovered from the enzymatic hydrolysis of RPI (Degree of hydrolysis (DH ) between 8.5 and 9% (DH1)) did not affect human fibroblast cells, inhibiting 83.9% of MCF-7 cells’ proliferation and showing a mass yield of 22.9% (based on the initial DRM). The SDS-PAGE gel revealed that DH1 was composed mainly of 10 kDa peptides and, to a lesser extent, 5 and 2 kDa. It is concluded that DH1 is a promising peptide extract for future research as a putative anti-breast cancer agent.

## 1. Introduction

Breast cancer is considered one of the main public health problems, and is the most frequent in women in developed and developing countries [1]. Although various medical procedures, including immunotherapy, radiation therapy, chemotherapy and others, have been used for decades in cancer treatment, current survival rates suggest that more definitive and effective treatment strategies are required [2].

Extensive research revealed that food-derived protein hydrolysates can be important for cancer treatment, thus explaining the significant attention that this research area currently attracts [3,4,5,6,7,8,9]. Peptides are compounds more bioavailable than proteins or free amino acids [10]. Therefore, they possess critical advantages as alternative chemotherapy, such as their high affinity, good penetration in tissues, strong specificity for targets and low toxicity [11].

Bioactive protein hydrolysates or peptides can be obtained from different food protein sources [12]. Rapeseed (*Brassica napus* L.) is one of the most important oil crops cultivated on multiple continents, contributing more than 15% of the world’s edible oil supply [13]. The production of rapeseed oil involves the generation of large quantities of rapeseed meal as a by-product, which is undervalued and used mainly as animal feed [14]. According to Ivanova et al., 2017 [12], rapeseed meal may reach up to 48% of the mass of the processed seeds. Due to its high protein content (38–48%) and relatively balanced amino acid composition, DRM could be used as a high-protein raw material in the production of bioactive peptides [12]. The main proteins in DRM are Cruciferin and Napin. Cruciferin has an average molecular mass of around 300 kDa in its native conformation, and fully dissociates into six subunits; each one comprises two polypeptides chains of approximately 30 and 20 kDa, linked by a disulfide bond. At the same time, Napin has a molecular weight between 12.5 and 14.5 kDa and comprises two polypeptide chains linked by two disulfide bonds, of 4.5 kDa and 10 kDa [15].

Different processes have been developed to obtain bioactive peptides from rapeseed. These have shown antiproliferative activity in different types of cell lines, such as HeLa, HepG2 and MCF-7. In the first case, a protein isolate from rapeseed meal was obtained using a saline solution, and antiproliferative peptides were subsequently recovered on HeLa cells with the enzymes Alkalase 2.4 L and Flavourzyme (cell inhibition: 40%, recovering 37% of total protein in HDR flour according to a Bradford test) [16]. In the second case, peptides with anticancer activity on HepG2, HeLa and MCF-7 cells were obtained by fermentation in solid-state with a mixture of bacterial and enzymatic synergy (cell inhibition: 36.97, 25.23 and 30.62%, respectively) [5]. This treatment was also used to obtain peptides with antiproliferative activity on MCF-7 cells, although they showed a low inhibition (1.26 ± 4.10%) and yield of rapeseed peptides (12.568 ± 0.236%) [17]. Therefore, low yields and low proliferative activities have been obtained, and their cytotoxic effect on healthy cells has not been evaluated. The alkaline extract (RAE) was evaluated as a substrate to demonstrate that a higher protein purity in the substrate leads to a higher MCF-7 inhibition.

The objective of the present study is to develop a process to obtain an enzymatic hydrolysate of rapeseed protein, which inhibits a breast cancer cell line but not healthy human fibroblasts, from defatted rapeseed meal using a commercial protease, promoting new uses for products derived from sub-valorized agro-industrial rapeseed products.

## 2. Materials and Methods

### 2.1. Raw Materials

Rapeseed cake (*Brassica napus*) was donated by Molinera Gorbea (Temuco, Chile). The agroindustrial residue was milled into flour using an IKA A-11 Basic Analytical mill. After, it was defatted with petroleum ether for 48 h, and then it was filtered, dried under the hood and stored in sealed containers. The MCF-7 breast cancer cell line was obtained from ATCC, VA, USA (ATCC^®^ HTB-22™), and gingival fibroblast cells—obtained as primary cultures—were a kind donation from Fernando Albornoz at Inbiocriotec SA, otherwise would be discarded, and were fully anonymized for their use, which was approved by the Institutional Ethics Committe of Universidad de Valparaíso to be used without informed consent (Act Nº CEC206-19). The commercial protease, Alkalase 2.4 L FG (endoproteinase from *Bacillus licheniformis*), was supplied by Novozymes (Bagsværd, Denmark). The enzyme had an activity of 694.5 U/mg, obtained using the azo-casein method [18]. Thermoase PC10F (*Bacillus stearothermophilus)* was supplied by Amano (activity of 269.4 U/mg using the azo-casein method).

### 2.2. Defatted Rapeseed Meal Characterization

The following analyses (dry matter, protein, crude fiber, fat and ash) before and after enzyme treatment of DRM were performed according to the methods described in AOAC (1990) [19]. Dry matter was determined by weight loss after drying (60 °C for 24 h). Protein content was determined indirectly from the total amount of nitrogen, measured by Kjeldhal’s method, multiplied by the factor 5.53 [20]. Fat content was determined by the Soxhlet extraction of lipids from dry samples, using petroleum ether as the solvent [19]. Ash content was determined gravimetrically after ashing at 550 °C for 6 h [19].

### 2.3. Obtaining of Protein Isolate (RPI)

DRM was suspended in sodium hydroxide 0.1 (M), pH 12.5, solid/liquid ratio of 1:10 (*w*/*v*), and incubated for 1 h at room temperature [21] on a magnetic stirrer. It was then centrifuged (5000 rpm, 15 min, 4 °C; ThermoFisher Waltham, MA, USA). The remnant solid was treated twice under the same conditions. The three supernatants were combined (alkaline rapeseed extract (RAE)) with the meal residue, dried at 35 °C and stored at room temperature (18 °C). Subsequently, the RAE was adjusted to pH 4.5 to precipitate proteins (with 5 M HCl) and was centrifuged for 30 min at 5000 rpm. The pellet (rapeseed protein isolate (RPI)) was resuspended in distilled water, pH 8, while the supernatant (SA) was freeze-dried and stored at room temperature (18 °C).

### 2.4. Enzymatic Hydrolysis

RPI was hydrolyzed with Alkalase 2.4 L and Thermoase PC10F. The enzymatic hydrolysis was carried out in batches in a 50 mL reactor using the pH-Stat method. A measured amount of the RPI substrate (15 mg/mL) (in terms of protein content, N = 5.53) was suspended in deionized water. The protein solution was adjusted by adding 1 N NaOH to a pH of 8 and a temperature of 50 °C. A measured amount of Alkalase 2.4 L was previously suspended in phosphate buffer (100 mM) at pH 8 to obtain an enzyme/protein ratio of 3 and 5% (*w/w* (wet weight of the enzyme preparation/dry weight of protein on the substrate)). For Thermoase PC10F, the same conditions of pH, temperature and E/S ratio were used. The RAE (9 mg/mL) hydrolysis was carried out with Alkalase 2.4 L (pH 8, 50 °C, E/S 3 and 5% *w/w*). The direct hydrolysis of DRM was carried out with a substrate concentration of 20% *w/v* (30.5 mg/mL) at pH 8 for 3 h (E/S 3, 5 and 10% *w/w*; enzyme wet matter/substrate protein dry matter). The protein concentrations used in the substrates (RPI, RAE, DRM) were different in order to evaluate how higher non-protein content influences the antiproliferative activity of MCF-7 and healthy human fibroblast cells. Control experiments were also performed without the addition of the enzyme. Hydrolysis reactions were stopped by heating the reaction mixture at 85 °C for 10 min. The hydrolysates were then frozen at −80 °C and freeze-dried. The degree of hydrolysis was determined by the modified OPA method [22]. The following hydrolysates were selected: C (control), DH0 (without hydrolysis (time 0)), DH1 (hydrolysis 8.5–9.0%), DH2 (hydrolysis 14.5–15.5%).

### 2.5. Antiproliferative Activity

The effect of the peptide extracts on the antiproliferative activity of MCF-7 and human fibroblast healthy cells was measured using the resazurin assay. This method is based on the ability of viable cells to reduce this molecule to form a fluorescent product with an excitation wavelength at 544 nm and an emission at 590 nm. Although it is based on the measurement of the viability of the cells, it is widely used as a determination of cell proliferation or survival, since each alive cell will contribute to the total fluorescent signal. Thus, the more cells, the higher the fluorescent signal. Survival was calculated from the ratio of relative fluorescence units (RFU) for cells exposed to the treatment and cells exposed to cell culture media, expressed as a percentage. The inhibition of cell proliferation was calculated by the difference between 100% and the survival percentage [23].

Therefore, to achieve our objective of evaluating the antiproliferative activity of peptide hydrolysates with different degree of hydrolysis (DH) values and substrates (RAE, PPI, DRM) on MCF-7 and human fibroblast, dried hydrolysates were suspended in DMEM culture media, and the cellular inhibition was studied using a range of 3.75–10 mg/mL.

### 2.6. SDS-PAGE Analysis

SDS-PAGE was performed as previously described [24] using low-molecular-weight markers (2000–250,000 Da, Bio Rad, Hercules, CA, USA). The electrophoresis was performed for 50 min at 120 mV, and the gels were stained following the Coomassie method [25].

### 2.7. Membrane Separation

The extract (DH1) was passed through ultrafiltration membranes with molecular weight cut-offs (MWCO) of 3 and 10 kDa, in an Amicon-stirred ultrafiltration cell (Millipore Corporation, Darmstadt, Germany). Finally, all the fractions were lyophilized and saved in a desiccator until use.

### 2.8. Statistical Analysis

Statistical analysis was performed using Minitab (Version 18, Minitab Inc, State College, PA, USA), and a significant difference was determined with a 95% confidence interval (*p* < 0.05) using one-way analysis of variance (ANOVA) followed by Tukey’s method for the paired comparison of samples.

## 3. Results

### 3.1. Process Mass Balance and Characterization of the Recovered Extracts

**Figure 1:**
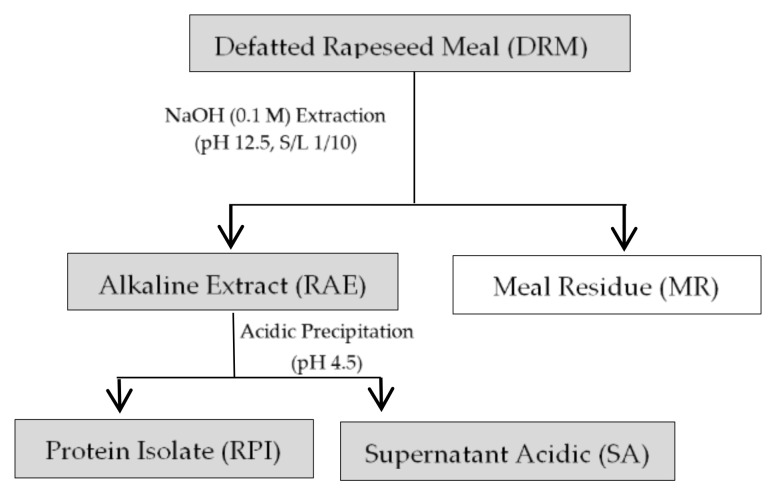
Protein extraction workflow.

The workflow of protein extract extraction is shown in Figure 1, and the proximate composition (g/100 g wet matter) of DRM is shown in Table 1. These values are well correlated with those reported in the literature [26,27,28]. As expected, DRM presented high protein content; thus, DRM proteins can be used as a potential raw material for bioactive peptide production. Its low-fat content offered an effective previous treatment with petroleum ether because the initial meal contains 11% fat. The defatting stage favors the protein extraction stage because it destabilizes the rigid cell wall of the DRM, and this facilitates the permeation of the solvent for the solubilization of proteins [29].

From 30.6% in DRM, the protein is concentrated to 42.7% in RAE due to the alkaline solubilization of DRM proteins, and 72.1% in RPI after isoelectric precipitation of the proteins contained in RAE. The yield of protein recovered in RAE was 76.5%, while for RPI/RAE it was 66.2% and for RPI/DRM it was 50.7%. This protein recovery data coincide with the report by Pedroche et al. (2004) [28]. Other authors obtained a DRM protein isolate by the isoelectric precipitation of alkali-extracted proteins, which contained a higher amount of crude protein (86.9% ± 0.0) and a relatively low amount of non-protein compounds [12]. The higher protein yield, compared to this research, could be due to differences in the process, such as a DRM treatment with ethanol before alkaline treatment to remove phenols, a different DRM/solvent (NaOH) ratio, and the incubation time with the solvent. In other reports, Gerzhova et al. (2016) [26] obtained a protein yield of 58.1% at pH 12 and an S/L ratio of 15% *w/v*. On the other hand, those reported by Das Purkayastha et al. (2015) [30], who applied a response surface methodology approach to optimize conditions, reported protein yields in the range of 35–46% at pH 11 [30]. Therefore, the alkaline solubilization of DRM and the isoelectric precipitation of proteins was identified as an efficient method for the recovery of proteins and the separation of other components, such as ash, fibers and carbohydrates, and subsequently, the enzymatic hydrolysis conditions were studied.

### 3.2. Protein Hydrolysates Production

**Figure 2:**
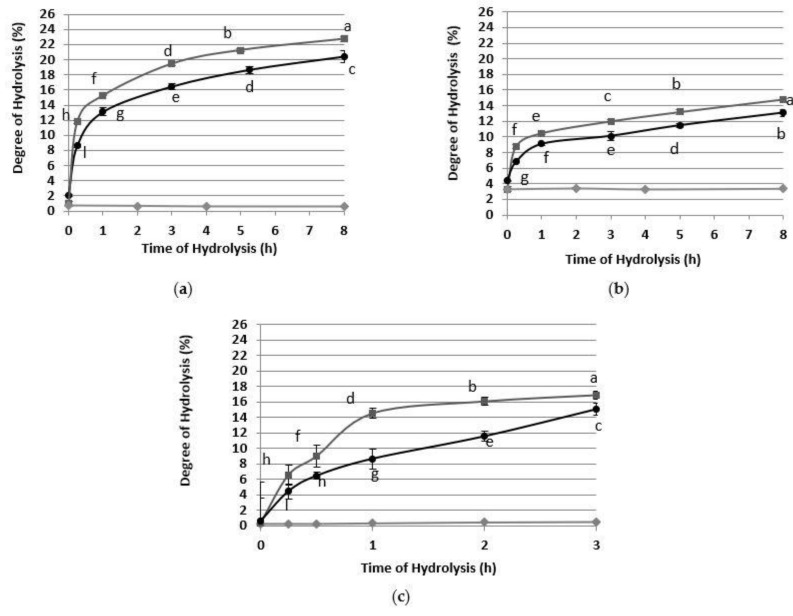
Effect of enzyme/substrate ratio (E/S) on the degree of hydrolysis with Alkalase 2.4 L FG (50 °C, pH 8). (**a**) RPI as substrate, (**b**) RAE as substrate, (**c**) DRM as substrate. Control without enzyme (◆), E/S = 3% (●), E/S = 5% (■). E/S = wet weight of enzyme preparation/dry weight of protein of the substrate. The degree of hydrolysis was measured by the OPA method. Mean ± SD (*n* = 4). The same letter means there is no significant difference (*p* < 0.05), 95% confidence level. The comparisons were made among all the observations.

Protein substrates were hydrolyzed using Alkalase 2.4 L FG, a well-known non-specific endoprotease widely used in food industry processes [31]. As shown in Figure 2, comparing the three controls, the RAE presented a higher initial DH (Figure 2b), which could be due to a greater number of peptides hydrolyzed by the NaOH used for the solubilization of proteins. Additionally, the controls do not vary over time, indicating no instability or hydrolysis due to process temperature, pH, etc. The hydrolysis rates were fast in the initial stage, and then gradually decreased. Valencia et al. (2014) [18] obtained the same behavior in the hydrolysis of fish protein with Alkalase 2.4 L, and they concluded that the decrease in the reaction rate was due to an inhibition by the hydrolysis product, and not to an enzyme inactivation or substrate exhaustion. Additionally, DH was increased in all cases by increasing the enzyme concentration from 3 to 5%. As shown in Figure 2, at E/S 5%, the RPI substrate obtained a higher DH (22%) compared to RAE (15%) and DRM (17%) (Figure 2a–c). In the case of RAE and DRM, the solubilization of other non-protein components such as carbohydrates could have interfered with the enzyme’s catalytic action. According to He et al. (2013) [32], it reached a maximum DH of 11.4 ± 0.1% at 4 h of DRM hydrolysis with Alkalase 2.4 L (E/S ratio 4%, based on the protein content of RPI) [32]. These authors suggested that RPI may be more resistant to Alkalase 2.4 L proteolysis than other proteins, such as cowpea and pea, and that therefore a low DH is achieved. However, in our case, the enzyme could be inactivated as a result of the hydrolysis products. Furthermore, in the case of RAE, its lower DH was related to its lower protein concentration in the substrate.

### 3.3. Inhibition of MCF-7 and Human Fibroblasts Proliferation by the Protein Hydrolysates

#### 3.3.1. Inhibition of MCF-7 and Human Fibroblasts Proliferation by the Protein Hydrolysates Obtained with Alkalase 2.4 L from RPI and RAE

**Figure 3:**
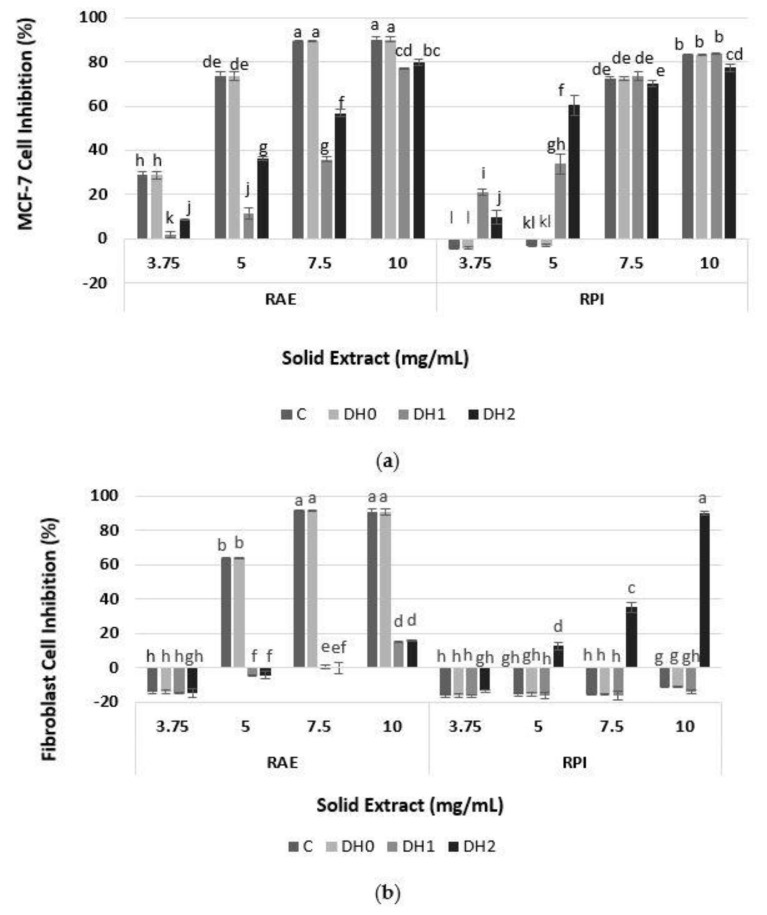
Cell inhibition of protein hydrolysates extracted from RAE and RPI (**a**) on MCF-7 cancer cell line, and (**b**) on healthy human fibroblast cell. Percentages of inhibition are calculated with reference to the signal of cells grown from extract-free cell culture media. C: control; DH0: without hydrolysis (time 0); DH1: hydrolysis 8.5–9.0%; DH2: hydrolysis 14.5–15.5%. Mean ± SD (*n* = 3). The same letter means there is no significant difference (*p* < 0.05), 95% confidence level.

The functionality of a hydrolysate is tied to the nature and the composition of the peptides generated during hydrolysis. The relationship between the DH of the peptides recovered and the antiproliferative activity on the MCF-7 and healthy human fibroblast was studied. As shown in Figure 3a, there was a concentration-dependent inhibition of MCF-7 proliferation for RAE and RPI at all hydrolysis degrees. Other studies with DRM are consistent with this result [5,17,33,34]. The results showed that RAE (C, DH0, DH2) and RPI (C, DH0, DH1) each had an inhibition equal to and greater than 80% of the MCF-7 proliferation at a 10 mg/mL concentration. In addition, RAE (C, DH0) inhibited with more than double the effectivity of its hydrolysates (DH1, DH2) at 7.5 mg/mL. This could be explained by the presence of smaller peptides in the RAE, which caused the decrease in the inhibitory effect observed in RAE hydrolysates after Alkalase 2.4 L FG treatment (DH1, DH2). This was supported additionally by the observation that the antiproliferative activity of the RAE (C) exposed to the same reaction conditions, without an enzyme, had a constant higher inhibitory activity of around 80% at concentrations above 3.75 mg/mL. Therefore, the Alkalase 2.4 L FG treatment resulted in a loss of RAE antiproliferative activity for all the concentrations, which might be because the non-protein compounds did not contribute to a great extent to the antiproliferative activity of RAE and its hydrolysates on MCF-7 cells. However, for RPI, the inhibition of cell proliferation among the C, DHO, DH1 and DH2 extracts was comparable at 7.5 and 10 mg/mL. Similar results were presented by Jamuna et al. (2017) [33], who demonstrated that the difference in the inhibitory effects of SKOV3 cancer cell proliferation between a soy protein (69%) and its hydrolysates (64%) was not significant. However, in this work the proteolytic hydrolysis with Alkalase 2.4 L FG improves the antiproliferative effect on the MCF-7 of RPI DH1 and DH2 at low concentrations.

A promising candidate for cancer treatment would be one that does not demonstrate an inhibition of healthy cell proliferation [35]. Therefore, as shown in Figure 3b, the antiproliferative activities of the same extracts on healthy human fibroblasts cells were studied, to select the ones that do not affect the viability of healthy cells. As observed, the only extracts that did not affect healthy cells’ proliferation were RAE (DH1 and DH2) (except at 10 mg/mL) and RPI (C, DH0 and DH1), at any extract concentration evaluated. Therefore, in this case, an effect of the substrate used and DH on cell inhibition of human fibroblast was demonstrated because some extracts and their hydrolysates were toxic to healthy cells. This could be due to the peptides’ sizes (preferably small, as will be discussed following the SDS-PAGE results) and the non-protein compounds solubilized with the proteins, such as carbohydrates, phenols and glucosinolates, which could affect human fibroblasts. Other researchers have used healthy mouse fibroblast cells (NIH3T3) to study their protein extracts’ antiproliferative capacities towards normal cells [33]. For example, Chi et al. (2015) [36] showed that a purified clam peptide (Trp-Pro-Pro) had strong cytotoxicity to HeLa cancer cell lines in a dose-dependent manner, but was not toxic in NIH3T3 cells from mouse fibroblasts. It is important to highlight that the selectivity exercise is more powerful if performed with human cells than mouse ones, because the MCF-7 cells are human and specifically with fibroblasts that are the more common healthy cells that an anticancer drug would interact with in a clinical scenario.

#### 3.3.2. Inhibition of MCF-7 and Human Fibroblasts’ Proliferation by the Protein Hydrolysates Obtained with Alkalase 2.4 L from DRM

**Figure 4:**
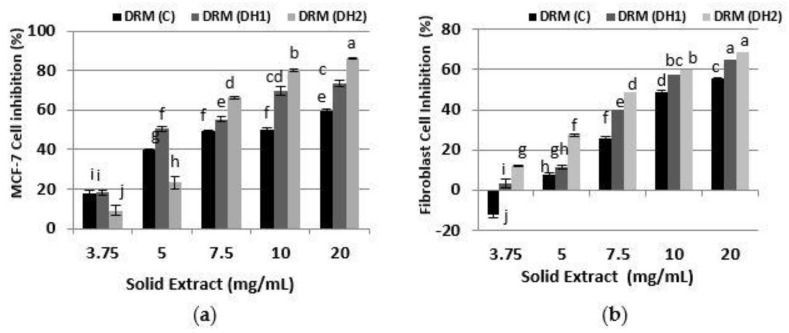
Cell inhibition of protein hydrolysates extracted from DRM. (**a**) on MCF-7 cancer cell line, (**b**) on healthy human fibroblast cell. Percentages of inhibition are calculated in reference to the signal of cells grown of extract-free cell culture media. Mean ± SD (*n* = 3). The same letter means there is no significant difference (*p* < 0.05), 95% confidence level).

Subsequently, the antiproliferative activity on MCF-7 and human fibroblasts was studied with hydrolysates obtained with the Alkalase 2.4 L treatment of DRM, as shown in Figure 4. DRM (C) showed a cellular inhibition of MCF-7 of 60% at 20 mg/mL, and 40–50% at concentrations between 5 and 10 mg/mL. This shows that the hydrolysis conditions (water at pH 8 and 50 °C, for 3 h) could solubilize compounds with antiproliferative effects on the MCF-7 cell line. Such compounds could be unhydrolyzed DRM proteins that demonstrated inhibitory effects on MCF-7 in RPI (C). Besides this, acid pectin and other components such as hemicelluloses could be solubilized. Similar results were obtained by Cobs-Rosas et al. (2015) [37], who indicated that the treatment with alkaline water (pH 8) of the DRM produced acid pectin that inhibited around 70% of MCF-7 cells in a concentration range of 5–20 mg/mL, with an inhibitory response not dependent on the extract dose.

Additionally, it was shown that treatment with Alkalase 2.4 L improved cellular inhibition on MCF-7. In this case, the effect of DH indicates that at high concentrations, DRM (DH2) showed a higher cellular inhibition of MCF-7 cells compared to DRM (DH1), reaching an antiproliferative activity of 86.3% and 80.4% at 20 and 10 mg/mL, respectively. Although at low concentrations (3.75–5 mg/mL), the inhibitory effect observed on MCF-7 was the opposite, the extract with DRM (DH1) was more effective. This latter could be due to the effect of small peptides, which may have probably been hydrolyzed or found in lesser amounts in the extract with DRM (DH2).

As shown in Figure 4b, the hydrolysates obtained with Alkalase 2.4 L from the DRM had a strong cytotoxicity on healthy human fibroblast cells, under all the concentrations studied. Furthermore, DRM (C) also affected human fibroblast cells at high concentrations. The latter may be due to the presence of soluble compounds in the control, such as pectic, hemicellulosic and lignocellulosic oligosaccharides, and antinutritional components (glucosinolates, lignin, phenols), which could be cytotoxic to human fibroblasts. This means that the composition and purity of the substrate is very important in order to obtain selectivity towards cancer cell lines.

### 3.4. Effect of the Type of Hydrolysate Obtained from RPI with Commercial Proteases on Cell Inhibition on MCF-7 and Human Fibroblast

**Figure 5:**
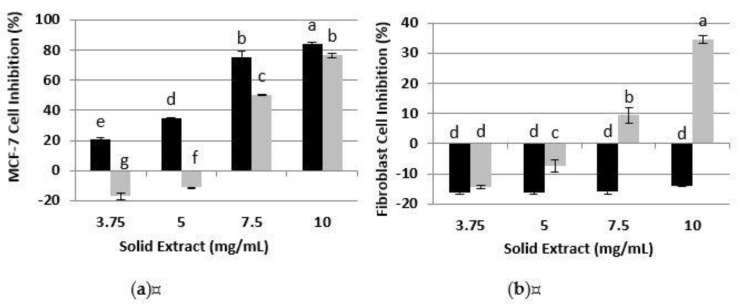
Cell inhibition of protein hydrolysates extracted from RPI with different commercial proteases: DH1 with Alkalase 2.4 L (■), DH1 with Thermoase PC10F (■). (**a**) On the MCF-7 cancer cell line; (**b**) on healthy human fibroblast cells. Percentages of inhibition are calculated with reference to the signal of cells grown with extract-free cell culture media. Mean ± SD (*n* = 3). The same letter means no significant difference (*p* < 0.05), 95% confidence level.

To compare the effect on cell inhibition in MCF-7 and human fibroblasts, two types of hydrolysates obtained from RPI, with two proteases with different mechanisms of action, were evaluated: Alkalase 2.4 L (serine protease) and Thermoase PC10F (metalloprotease). Hydrolysis was carried out with each enzyme under the same reaction conditions, and two hydrolysates were obtained with the same DH (DH1). DH1 was selected because it did not inhibit healthy human fibroblast cells under any concentration previously studied for those hydrolyzed with Alkalase 2.4 L, and at the same time, it was a potent inhibitor of MCF-7 cells. As shown (Figure 5a), the hydrolysate recovered with the Alkalase 2.4 L enzyme demonstrated greater cellular inhibition of MCF-7 both at high and low concentrations. In fact, the hydrolysate obtained with Thermoase PC10F did not inhibit breast cancer cells at concentrations less than 7.5 mg/mL. From a pharmacological perspective, cytotoxicity at lower concentrations is one of the most valuable properties for clinical application [37]. Therefore, the extract obtained with Alkalase 2.4 L is a promising anticancer agent, compared to Thermoase PC10F, with cellular inhibition levels of 82.9% at 10 mg/mL and 19.7% at 3.75 mg/mL. The observed effect of the type of hydrolysate could be due to the size (length and molecular weight) and sequence of the hydrolyzed peptides, depending on the enzymatic specificity. Jumeri et al., 2011 [38] obtained similar results with protein extracts from a solitary tunicate with Alkalase 2.4 L and Thermoase PC10F, presenting a greater antiproliferative effect (stomach (AGS), colon (DLD-1) and cervical cancer cells (HeLa)) than Alkalase 2.4 L hydrolysates. The authors indicate that this effect could be governed by the higher content of hydrophobic amino acids recovered with Alkalase 2.4 L FG compared to Thermoase PC10F. Furthermore, Alkalase 2.4 L is a less specific protease than Thermoase PC10F [38], the composition of the peptides could be more varied, and the type of peptide is more bioactive at low concentrations. The effect of the type of RPI hydrolysate on human fibroblast cell inhibition is presented in Figure 5b. The hydrolysate obtained with Thermoase PC10F inhibits fibroblast cells at high concentrations, while that obtained by Alkalase 2.4 L did not show toxicity under any of the concentrations studied. Therefore, an effect of the type of hydrolysate was displayed, obtained from the RPI with the above-mentioned proteases, which could be due to the amino acid composition of the hydrolysates and their molecular weights, since although the DH reached was the same, this does not indicate that the peptides formed have the same size and sequence, given the different catalytic mechanisms of the enzymes used.

### 3.5. Characterization of Protein Extracts Obtained with Alkalase 2.4 L by SDS-PAGE Electrophoresis

**Figure 6:**
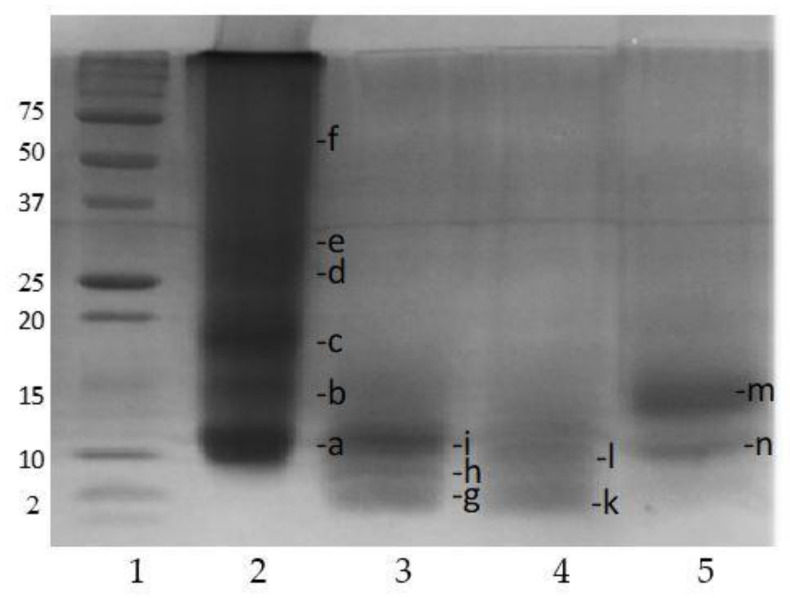
SDS-PAGE of protein isolate. Line 1: molecular weight marker; line 2: RPI; line 3: RPI (DH1); line 4: RPI (DH2); line 5: supernatant acidic (SA). The letters (a–n) represent the molecular weights (kDa) of each band in the extracts: a (10), b (15), c (18), d (25), e (30), f (50), g (2), h (5), i (10), k (2), l (8), m (15), n (10).

The characterization of protein extracts by SDS-PAGE electrophoresis, in order to evaluate the relationship between the sizes of the recovered peptides and the demonstrated inhibitory effects, is shown in Figure 6. RPI was mainly composed of low-molecular-weight fractions between 10 and 50 kDa (line 2) (a–f). This is in agreement with the results for canola protein isolates (mainly 14 to 59 kDa) presented by Wu et al. (2008) [39]. Supernatant acidic (SA) showed one band at 15 kDa of high intensity, and a second band, that was less pronounced, at 10 kDa (line 5). The SA analysis was done to determine the size of the DRM proteins that were precipitated compared to those that were soluble in SA. Therefore, the pronounced 15 kDa band in SA (m) could belong to Napin, as it is a strongly basic protein with an isoelectric point around 11 that does not precipitate at pH 4.5 [21] (pH at acid precipitation of the proteins extracted in this research). It is known that the characteristic bands of rapeseed proteins are between 18 (c) and 50 kDa (f) for Cruciferin, whereas for Napin they range between 5 and 15 kDa [27]. According to Hajirostamloo et al. (2010) [10], the RPI and SA (obtained from rape meal at pH 12 and precipitation at pH 4.5) were mainly composed of low molecular weight fractions between 5 and 33 kDa, but in different proportions, although a 53 kDa band was present only in RPI and not in SA. The 8 kDa protein band was the most abundant fraction in the SA, indicating that a possible breakdown of Napin may occur during rapeseed processing. In this research, RPI (DH1) (line 3) showed a more pronounced band at 10 kDa (i) and two other less intense bands at 5 (h) and 2 kDa (g), which become more intense in RPI (DH2) (line 4) (l and k). In the latter, the 10 kDa band disappeared. Therefore, it is demonstrated that the enzyme Alkalase 2.4 L hydrolyzed polypeptide chains greater than 10 kDa during the first 15 min of reaction (hydrolysis time in RPI (DH1)).

This study demonstrated that RPI (DH1), which inhibited MCF-7 cells by 83.9%, without affecting the human fibroblast cell viability, was mainly composed of 10 peptides, and to a lesser extent 5 and 2 kDa. The mass yield of this hydrolysate was 22.9% (based on the initial DRM).

### 3.6. Effect of the Mean Size of the Protein Extract RPI (DH1) on the Cellular Inhibition of MCF-7 Cells

The RPI (DH1) extract was separated into different peptide fractions (<3, 3–10 and >10 kDa) by membrane ultrafiltration.

It has been reported that short protein hydrolysate peptides generally exert a greater anticancer activity than large polypeptides [6]. After ultrafiltration, it was shown that 46.6% of the peptides were greater than 10 kDa, 36.3% had a molecular weight between 3 and 10 kDa, and 17.2% were less than 3 kDa (Table 2). Therefore, a higher percentage of peptides were greater than 10 kDa, followed by the fraction that were 3–10 kDa, and a lower percentage were less than 3 kDa. Considering the protein purity of the recovered fractions, the second fraction (3–10 kDa) turned out to be the purest, presenting 84.0% of proteins.

**Figure 7:**
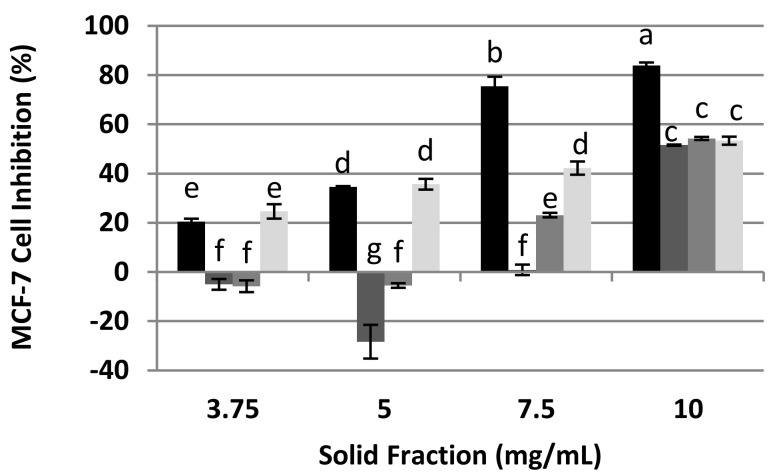
Effect of the molecular weight of the fractions obtained from the RPI (DH1) by ultrafiltration on the cellular inhibition of MCF-7. RPI (DH1) initial (■), fraction I (>10 KDa) (■), fraction II (3–10 KDa) (■), fraction III (<3 KDa) (■). Mean ± SD (*n* = 3). The same letter means no significant difference (*p* < 0.05), 95% confidence level.

The antiproliferative effect on MCF-7 of the fractions recovered in our study is shown in Figure 7. As seen, at 10 mg/mL, the three fractions inhibited about 50%, while unfractionated RPI (DH1) had 80% antiproliferative activity on the MCF-7 cells. This could be due to a synergistic effect between two or three of the evaluated peptide fractions, which results in a higher antiproliferative activity in the total hydrolysate. At a concentration of 7.5 mg/mL, fraction I (>10 kDa) did not show inhibition of MCF-7’s cell viability, and fractions II and III inhibited 23.1% and 42.2%, respectively, while the antiproliferative effect of RPI (DH1) was 75.5%. Fraction III (<3 KDa) was the only one that inhibited MCF-7 cells at concentrations less than 5 mg/mL. This indicates that small peptides are responsible for the inhibitory effect of RPI (DH1) between 3.75 and 5 mg/mL. This result is very significant, because it explains the difference between RPI (DH1) and RPI (DH0) at low concentrations, whereby, as seen previously, RPI did not show an inhibitory effect at concentrations lower than 5 mg/mL, while RPI (DH1) inhibited between 20 and 40% at the same concentration. Therefore, enzymatic hydrolysis with Alkalase 2.4 L FG improved the cellular inhibition of MCF-7 at low concentrations. However, other compounds (such as polyphenols, neutral sugars and isothiocyanates) could influence the inhibition of MCF7 proliferation and could generate synergy with other present peptides. The authors reported similar results [40,41]. This result is beneficial for the formulation of products with bioactivity, and in fact, in most cases, the objective final product in obtaining bioactive peptides derived from food is not usually a single peptide with a purity of 99%, because of the unacceptably high cost and poor performance that would be involved. Furthermore, formulating products with various peptides can address the solubility problem, while conferring the same bioactivity level [42].

It should be mentioned that it was assumed that the obtained fractions were not cytotoxic for healthy cells of human fibroblast, because the protein extract RPI (DH1) was not cytotoxic for these cells, as demonstrated previously.

## 4. Conclusions

The alkaline treatment and isoelectric precipitation of DRM allowed for recovering an RPI with a mass yield of 22.9% (based on the initial DRM) and a high antiproliferative activity on MCF-7, without cytotoxicity towards healthy human fibroblasts. The enzymatic hydrolysis of RPI with Alkalase 2.4 L improved cellular inhibition of MCF-7 at low extract concentrations. An effect of DH was demonstrated, with low DH being more effective in promoting the selectivity of the extracts towards cancer cells. The peptide fraction <3 kDa in RPI (DH1) was the only one that inhibited the proliferation of MCF-7 cells at low concentrations, compared to fractions composed of peptides greater than 10 kDa and between 3 and 10 kDa, although other non-protein compounds could be involved in the antiproliferative activity of RPI (DH1). An effect of the type of hydrolysate was also demonstrated, as the Alkalase 2.4 L hydrolysates were more effective in terms of selectivity towards cancer cells. Furthermore, it was demonstrated that enzymatic hydrolysis directly from DRM showed high cytotoxicity in human fibroblast cells, suggesting the importance of high-purity protein extracts, such as hydrolysates obtained from RPI. The results of this study indicate that it was possible to develop a process to obtain DRM peptides with high cellular inhibition on MCF-7, high yield, and without cytotoxic effects on human fibroblast cells, and that therefore they could be potential ingredients in functional and nutraceutical foods with antiproliferative properties in MCF-7 breast cancer cells.

## Figures and Tables

**Table 1:** Proximate composition (g/100 g wet matter) of DRM and the recovered extracts.

Component	DRM	RAE	MR	RPI	SA
Total solid (g)	100	54.8 ± 4.2	51.2 ± 2.3	21.5 ± 0.6	33.2 ± 2.2
Humidity (g)	6.0 ± 0.3	-	-	-	-
Crude protein (g)	30.6 ± 1.5	23.4 ± 2.7	7.2 ± 2.1	15.5 ± 1.5	7.9 ± 2.4
Ash (g)	6.0 ± 0.0	12.8 ± 0.5	5.2 ± 0.1	1.7 ± 0.4	11.1 ± 0.1
Crude fiber (g)	10.3 ± 1.5	2.5 *	7.8 ± 0.4	-	-
Crude fat (g)	0.3 ± 0.0	-	-	-	-
Nitrogen free extract (g)	46.8 ± 1.0	15.7 *	31.1 ± 1.1	-	-

* Difference between DRM and MR.

**Table 2:** RPI (DH1) fractionation by ultrafiltration and peptide recovery.

	Total Solid (mg)	Protein (mg)	Protein (% *w/w*) ^a^	Protein (% *w/w*) ^b^
Fraction I (>10 kDa)	633.6 ± 6.8	455.8 ± 5.2	46.6	71.9
Fraction II (3–10 kDa)	422.3 ± 5.8	354.8 ± 6.6	36.3	84.0
Fraction III (<3 kDa)	243.8 ± 7.5	168.1 ± 6.1	17.2	68.9

^a^ % mg protein/mg total protein in RPI (DH1) initial. ^b^ % mg protein/mg total solid in the corresponding fraction. Mean ± SD (*n* = 2).

## Data Availability

All the data available is in the manuscript.
